# Influence of multidimensional determinants on physical activity behavior: a perspective from health promotion and behavior change theories

**DOI:** 10.3389/fpubh.2026.1865453

**Published:** 2026-06-25

**Authors:** Guiming Zhang, Kai Chen, Guosheng Ding

**Affiliations:** School of Architecture and Planning, Hunan University, Changsha, Hunan, China

**Keywords:** behavior change theory, empirical econometric analysis, health promotion theory, multidimensional determinants, physical activity behavior

## Abstract

It is widely recognized that the provision of sports facilities plays an important role in shaping individuals' physical activity behavior. However, existing studies have often focused on isolated facility attributes such as facility density or accessibility, while paying limited attention to facility diversity and quality, and many have not been explicitly guided by health promotion or behavior change theories. To address these gaps, this study conducts a questionnaire survey in five representative cities in China to examine the associations between multidimensional determinants and physical activity behavior within a theory informed analytical framework. Specifically, facility type diversity, facility quantity, facility quality, and facility accessibility are examined as core explanatory variables, together with individual characteristics, social environment factors, and health awareness, in relation to exercise frequency, exercise intensity, and exercise duration. Guided by health promotion theory and behavior change theory, the study applies descriptive analysis, correlation analysis, regression analysis, heterogeneity analysis, and robustness tests. The results indicate that: (1) facility type diversity, facility quantity, and facility quality are significantly and positively associated with exercise frequency, exercise intensity, and exercise duration, whereas facility accessibility shows a significant negative association with these activity behavior indicators; (2) with respect to individual characteristics, older age is associated with lower exercise participation, while males, civil servants, retirees, and individuals with higher income and educational attainment exhibit higher levels of physical activity; (3) in terms of the social environment, a supportive family exercise atmosphere and abundant community sports activities significantly promote people's physical activity behavior; and (4) regarding health awareness, its effect on activity behavior is particularly strong, with individuals who have greater health awareness being more actively engaged in physical activity.

## 1 Introduction

In contemporary society, health issues have attracted increasing public attention and have become an important indicator of the level of development of a region and even a country. With rapid economic growth and substantial improvements in living standards, people's pursuit of health has intensified, accompanied by a gradual rise in health awareness (1–3). In response, many countries have formulated national-level strategic health policies. For example, the United States' Healthy People 2030 sets clear objectives for health promotion and disease prevention over the next decade, including increasing levels of physical activity (4), while Japan's Health Japan 21 incorporates the extension of healthy life expectancy and the promotion of physical activity into its nationwide health action plan (5). China's Healthy China 2030 Plan Outline explicitly emphasizes strengthening the construction of community-based multifunctional sports facilities and fitness trails, aiming to establish a three-tier public sports facility network at the county, township, and village levels by 2030, and to achieve full coverage of a “15-min exercise circle" in urban communities (6, 7).

Under this context, public sports facilities, as essential physical infrastructure for supporting public health, are particularly critical in their rational allocation and effective provision. Specifically, as the material foundation for engaging in physical activity, sports facilities are closely associated with individuals' activity behavior. Existing studies indicate that the density of public sports facilities is positively correlated with the rate of physical fitness compliance within communities, and that well-planned and adequately equipped sports facilities can effectively promote physical health (8, 9). However, this stream of research tends to emphasize aggregate indicators such as facility density, providing limited insight into how different facility attributes jointly shape physical activity behavior. In addition to their health-related benefits, sports facilities also serve important social functions by facilitating social interaction among residents and strengthening community cohesion (10, 11), yet the behavioral pathways linking these social functions to individual activity decisions are often not explicitly examined.

A growing body of literature has examined the relationship between sports facility configuration and physical activity behavior. For instance, Yang et al. (12) found that diversified sports facilities can better meet the exercise needs of different population groups and increase participation in physical activity. While this study highlights the role of facility diversity, other key attributes such as facility quality and accessibility receive less attention. Similarly, Lee et al. (13) showed that an increase in the number of sports facilities is positively associated with physical activity by improving convenience and reducing crowding. Nevertheless, facilities are largely treated as homogeneous units, and functional differences across facility types are not fully considered. Moreover, Mohd Aznan et al. (14) emphasized the importance of facility quality in enhancing exercise experience and reducing injury risk, but its analysis mainly focuses on perceived quality and does not examine interactions with facility quantity or spatial accessibility.

Beyond physical infrastructure, previous studies have also recognized the importance of individual characteristics in shaping physical activity behavior. Box et al. (15) identified substantial differences across demographic groups, while Ashe et al. (16) observed that older adults tend to engage more frequently in physical activity due to greater leisure time. Although these findings provide valuable demographic insights, individual characteristics are often examined in isolation, with limited consideration of how personal attributes interact with environmental conditions. Time and economic constraints have also been identified as barriers to physical activity (17), yet these constraints are typically treated as control variables rather than core behavioral mechanisms within an integrated framework.

Social environmental factors constitute another important dimension influencing physical activity behavior. Sabo and Hunter Revell (18) demonstrated that family exercise habits and attitudes can foster a supportive environment for physical activity, while Noroozi et al. (19) showed that community-level facilities and organized activities can enhance residents' motivation to exercise. However, these studies generally focus on single social dimensions and do not jointly model social environment, individual health awareness, and facility conditions. Health awareness and personal interests have also been shown to influence physical activity behavior (20, 21), but such psychological factors are frequently examined independently from objective facility characteristics.

Overall, although pre-existing studies have generated substantial empirical evidence, several limitations remain. First, much of the literature is largely empirical and descriptive, focusing on isolated determinants, most commonly facility density or quantity, rather than adopting a comprehensive perspective that incorporates multiple facility attributes, including diversity, quality, and accessibility (22–24). Second, non-facility determinants such as individual characteristics, social environment, and health awareness are often analyzed separately, without being systematically integrated into a theory-based analytical framework. As a result, existing studies tend to identify statistical associations but provide limited explanation of the underlying behavioral mechanisms driving physical activity participation.

To address these gaps, grounded in health promotion theory and behavior change theory, this study develops a theory-informed framework to examine how multidimensional determinants jointly influence people's physical activity behavior. Specifically, sports facility allocation (diversity, quantity, quality, and accessibility) is conceptualized as a key environmental enabling factor, while individual characteristics, social environment, and health awareness are incorporated as motivational and behavioral determinants. Using questionnaire survey data from five representative Chinese cities, this study employs descriptive analysis, correlation analysis, regression analysis, heterogeneity testing, and robustness testing. By integrating facility-related and non-facility factors within a coherent theoretical framework, this study extends prior empirical research and provides policy-relevant evidence for optimizing sports facility allocation and promoting public physical activity.

The rest of this paper is organized as follows. Section 2 elaborates on health promotion theory and behavior change theory. Section 3 presents the detailed research design, including research sites, variable selection, and model construction. Section 4 conducts an in-depth empirical analysis and summarizes the questionnaire results. Section 5 draws conclusions.

## 2 Research framework and theoretical basis

**Figure 1:**
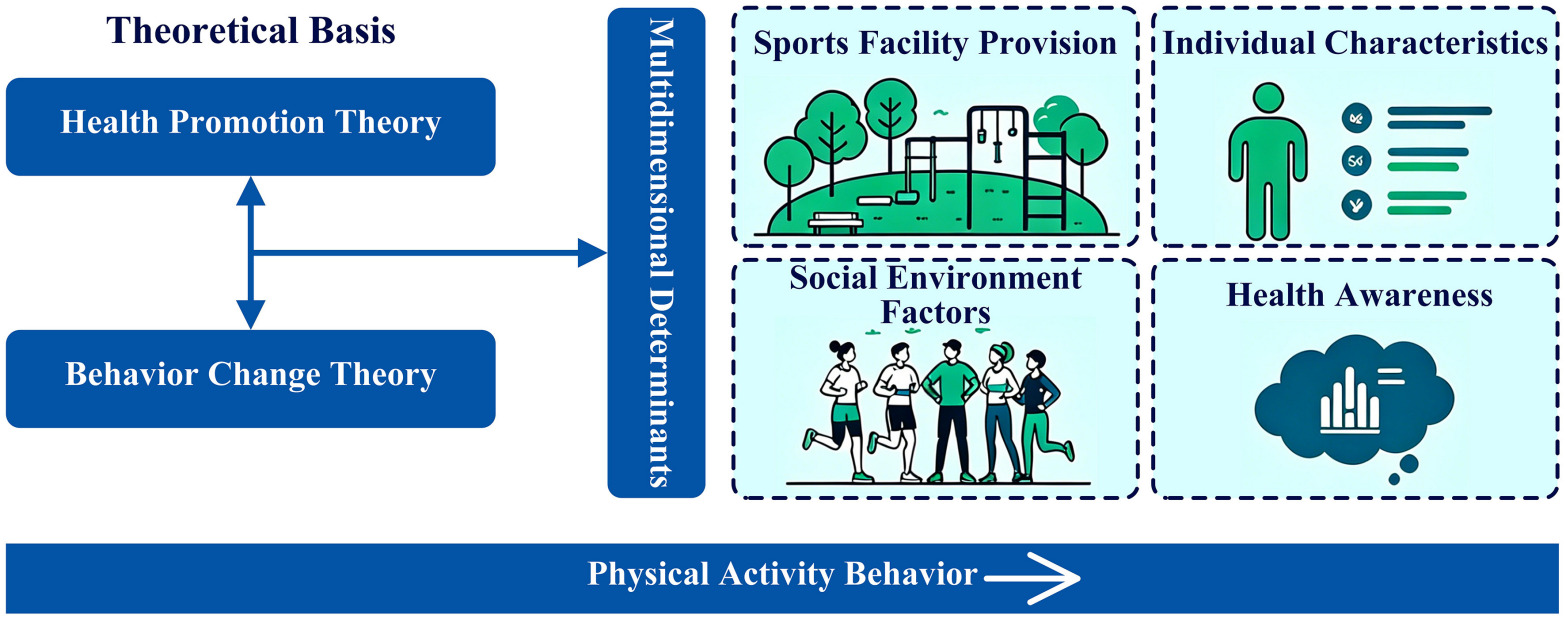
Framework for multidimensional determinants of physical activity behavior.

The whole framework for multidimensional determinants of physical activity behavior is shown in Figure 1. Guided by health promotion theory and behavior change theory, the research question of this study focuses on how multidimensional characteristics of sports facilities including facility type diversity, facility quantity, facility quality, and facility accessibility, together with individual characteristics, social environment factors, and health awareness, influence individuals' physical activity behavior as reflected by exercise frequency, exercise intensity, and exercise duration. Prior to presenting the empirical results, this section introduces the theoretical foundations of these two theories.

It should be emphasized that the selection of health promotion theory and behavior change theory is based on their complementary roles in explaining physical activity behavior. health promotion theory highlights how supportive environments, such as the availability, diversity, quality, and accessibility of sports facilities, facilitate individuals' participation in physical activity. In contrast, behavior change theory emphasizes that individuals may respond differently to similar environmental conditions due to differences in personal characteristics, social context, and health awareness. Integrating these two perspectives enables the study to link facility provision with heterogeneous behavioral outcomes, including exercise frequency, intensity, and duration.

### 2.1 Health promotion theory

The concept of health promotion first appeared in the public health literature in the 1920s (25). Health promotion is a multidimensional concept involving multiple levels of action. It focuses on the health of the entire population rather than targeting specific diseases or high-risk groups, with the overarching aim of improving overall population health. Moreover, it emphasizes the diverse determinants of health, extending beyond biological factors to include lifestyle behaviors, social environments, and socioeconomic conditions. Effective implementation of health promotion requires the active participation and collaboration of multiple stakeholders, including governments, social organizations, communities, families, and individuals, who must work together to advance health promotion initiatives (26, 27).

In terms of strategic approaches, the Ottawa Charter proposed three core strategies: advocacy, empowerment, and coordination (28). Advocacy aims to raise awareness of the importance of health among policymakers at all levels, social sectors, and the general public through communication and public engagement, thereby mobilizing collective efforts to promote health. Empowerment focuses on capacity building by disseminating health knowledge and developing health-related skills, enabling individuals and communities to prevent disease and promote health more effectively. It also involves enhancing individuals' and communities' ability to utilize health policies and healthcare services, thereby improving health literacy and self-care capacity. Coordination reflects the complexity of health determinants and highlights the need for close collaboration between the health sector and other social sectors to jointly address health-related issues and foster a supportive social environment for health promotion.

In the present study, health promotion theory provides an important theoretical foundation. From this perspective, sports facility provision is a key environmental determinant of health behavior, directly shaping individuals' opportunities, motivation, and constraints for physical activity. Specifically, facility diversity and quantity expand available exercise opportunities and increase the likelihood of matching different users' preferences, thereby facilitating participation in physical activity. Facility quality enhances user satisfaction, improves exercise experience, and reduces perceived safety risks, which in turn strengthens individuals' willingness to engage in regular physical activity. Facility accessibility reduces spatial and temporal barriers, lowers the time and effort costs of participation, and thus increases the feasibility of engaging in physical activity.

From a health promotion perspective, the rational planning and equitable distribution of sports facilities contribute to the creation of supportive environments that encourage active lifestyles. For instance, aligning facility types and spatial layouts with community characteristics and residents' needs enhances the convenience and utilization of such facilities, reflecting the strategy of creating supportive environments advocated in health promotion theory. Furthermore, increasing public awareness and utilization of sports facilities through information dissemination and promotion aligns with the advocacy and empowerment strategies of health promotion. Consistent with the emphasis on intersectoral collaboration, the development and improvement of sports facilities require coordinated efforts among governments, communities, and other stakeholders to effectively promote population health.

### 2.2 Behavior change theory

Behavior change theory plays a critical role in explaining and guiding changes in human behavior and provide important theoretical insights into the mechanisms underlying physical activity behavior. Commonly used frameworks include the knowledge-attitude-practice (KAP) model and the health-belief-model (HBM) (29, 30), both of which conceptualize behavior change from different perspectives by emphasizing the cognitive and psychological processes involved.

The KAP model posits that knowledge forms the foundation of behavior change, attitudes or beliefs serve as the motivational force that transforms knowledge into action, and practice represents the observable manifestation of knowledge and beliefs. In the context of physical activity, individuals who understand the health benefits of exercise, such as improved physical fitness, disease prevention, and enhanced mental well-being, and who develop strong beliefs regarding its importance are more likely to translate such knowledge into sustained physical activity (31). However, the application of the KAP model faces practical limitations, as individuals differ in their educational background, cognitive capacity, and life experiences. Consequently, some individuals may possess adequate knowledge about physical activity but still fail to engage in exercise due to constraints such as limited time, lack of skills, or insufficient access to appropriate facilities.

The HBM emphasizes individuals' perceptions of health threats, perceived benefits and barriers of health-related behaviors, and self-efficacy as key determinants of behavior change (30, 32). Individuals are more likely to engage in physical activity when they perceive themselves to be at risk due to physical inactivity, believe that exercise can effectively reduce such risks, and have confidence in their ability to overcome potential barriers, such as time constraints or physical fatigue. Nevertheless, the model is subject to limitations, as perceptions of health risks and exercise-related benefits can be influenced by factors such as risk preferences, sociocultural context, and information exposure. In some cases, individuals may underestimate health threats or misjudge the benefits and barriers of physical activity, thereby hindering behavior change.

Taken together, behavior change theories offer a multidimensional framework for understanding physical activity behavior by highlighting the combined influence of knowledge, beliefs, perceptions, and self-efficacy on individuals' exercise decisions and maintenance. In the context of this study, these theories help explain how sports facility provision functions as an external environmental trigger that shapes individuals' psychological processes and subsequent physical activity behavior. Specifically, facility diversity and quantity increase perceived behavioral opportunities and strengthen outcome expectations by providing more accessible and suitable exercise options. Facility quality enhances perceived safety and exercise satisfaction, which improves positive attitudes toward physical activity and strengthens behavioral intention. Facility accessibility reduces perceived effort and time costs, thereby lowering perceived behavioral barriers and enhancing self-efficacy.

Moreover, these theories provide a theoretical basis for intervention strategies that integrate facility provision with educational and motivational approaches, thereby promoting sustained physical activity and improving population health.

## 3 Research design and methodology

### 3.1 Data sources

**Figure 2:**
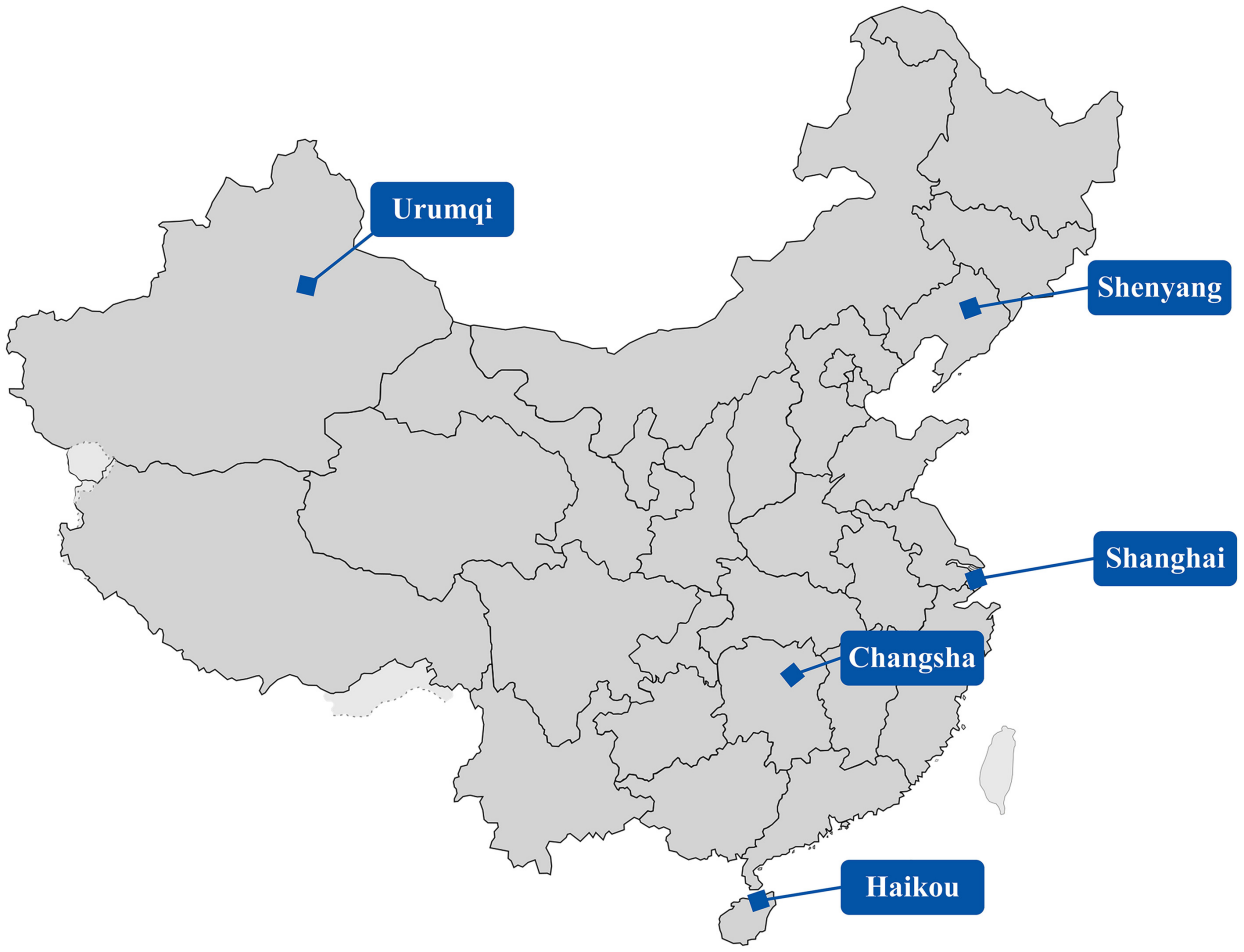
Spatial distribution of the sample cities in China.

To ensure the representativeness of the sample, this study follows a multi-stage stratified sampling design combined with random sampling procedures. Considering that regional differences in economic development, population density, urbanization level, climatic conditions, and cultural context may influence both the provision of sports facilities and individuals' physical activity behavior, five representative cities are first selected through purposive stratification across China, covering the eastern, western, southern, northern, and central regions. Specifically, Shanghai represents highly developed coastal metropolitan areas, Urumqi reflects cities in the western region with relatively sparse population and distinct ethnic and cultural characteristics, Haikou represents southern coastal cities with favorable climatic conditions, Shenyang captures traditional industrial cities in northeast China, and Changsha represents rapidly developing cities in central China. Together, these cities capture substantial regional variation in socioeconomic development and urban environments, thereby enhancing the representativeness and heterogeneity of the sample. The spatial distribution of the sample cities is illustrated in Figure 2.

In the second stage, within each selected city, 20 communities are randomly selected from different urban districts to ensure spatial coverage and avoid clustering bias, resulting in a total of 100 communities. In the third stage, respondents within each community are selected using simple random sampling, and 20 questionnaires are distributed per community to ensure consistency in sample allocation across regions. A total of 1,800 valid questionnaires are collected, yielding an effective response rate of 90.

The sample covers individuals from diverse demographic backgrounds. Specifically, males accounted for 52% of the respondents and females for 48%. Participants ranged in age from 18 to 70 years, with all age groups adequately represented. Occupational categories included civil servants, enterprise employees, self-employed individuals, retirees, and students. Income levels are classified into low-, middle-, and high-income groups based on local average income standards, with a relatively balanced distribution across categories.

The primary data source of this study is a questionnaire survey. Drawing on well-established measurement scales from both domestic and international literature, and in accordance with the objectives of this research, a structured questionnaire is developed. The questionnaire covers respondents' basic demographic characteristics (gender, age, income, occupation, and educational attainment), physical activity behavior (exercise frequency, exercise intensity, and exercise duration), sports facility characteristics (facility type diversity, facility quantity, and facility quality), family and community exercise environment and atmosphere, as well as individual health awareness. A combination of Likert-scale items, single-choice questions, and multiple-choice questions is employed to accommodate different types of data. During data collection, a multi-stage sampling strategy combining stratified sampling and random sampling is adopted to further enhance sample representativeness.

### 3.2 Variable definition

**Figure 3:**
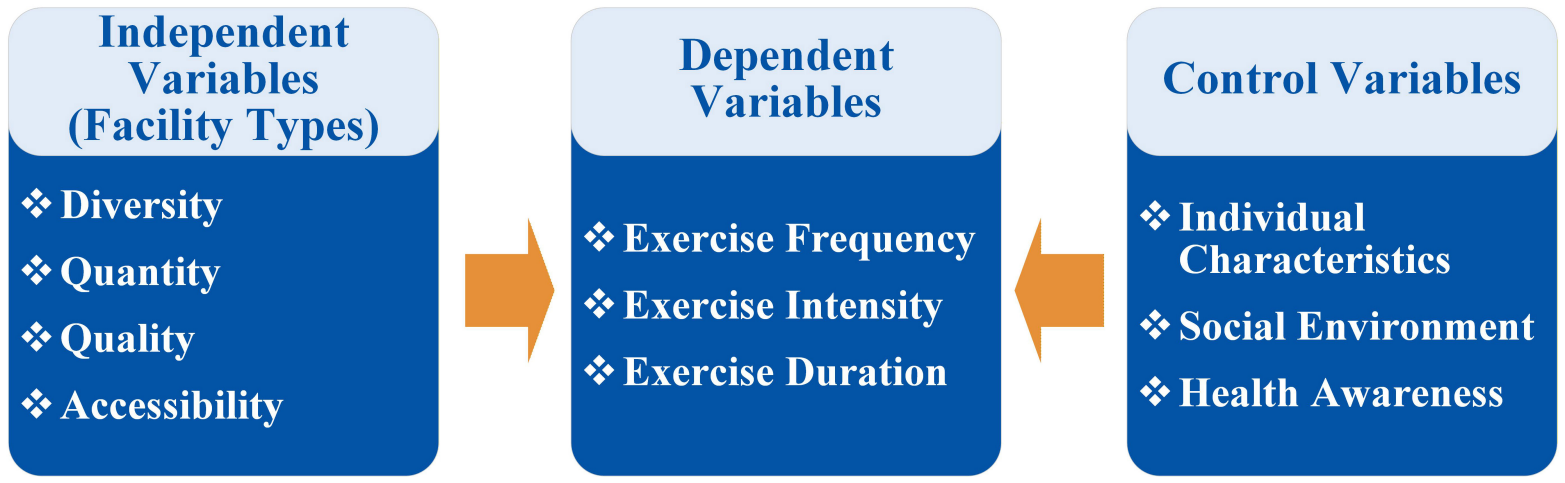
Definitions of independent variables, control variables, and dependent variables.

This study comprehensively analyze the combined effects of multiple independent variables of facility types and control variables on the physical activity behavior (i.e., dependent variables). Specifically, as shown in Figure 3, the independent variables consist of facility-related attributes, including facility diversity, quantity, quality, and accessibility, the control variables include individual characteristics, social environment factors, and health awareness, and the dependent variables measure physical activity behavior, operationalized in terms of exercise frequency, intensity, and duration, under study. Details are elaborated as follows.

#### 3.2.1 Independent variable definition

Facility-related configuration variables constitute the key independent variables in this study and mainly include the following dimensions.

Facility diversity is used to measure the variety of sports facility types available within a community or region. This indicator is constructed by counting the number of distinct types of sports facilities, such as ball courts (e.g., basketball, football, and tennis courts), fitness equipment (e.g., aerobic and strength-training equipment), recreational trails, and swimming pools. For example, if a community provides five different types of sports facilities, the facility diversity score is recorded as 5. This variable reflects the extent to which sports facilities can meet people's diverse exercise needs, as a wider range of facility types is more likely to attract individuals with different interests to participate in physical activity.

Facility quantity refers to the actual number of sports facilities within a community or region, including the number of fitness equipment units and sports venues. For example, a community with 20 pieces of fitness equipment and three sports venues is coded accordingly as a continuous variable. Facility quantity directly affects the convenience and availability of facility use, as a sufficient number of facilities can reduce waiting time and increase people's willingness to engage in physical activity.

Facility quality is measured using a multi-item Likert-scale construct adapted from previous studies on built environment and public facility evaluation. Respondents assessed facility condition, safety, comfort, and technological level on a five-point scale (1 = very poor, 5 = very good). The final facility quality score is calculated as the average of all items. Reliability and validity tests were conducted to ensure measurement robustness. The Cronbach's alpha value exceeds 0.70, indicating acceptable internal consistency, and confirmatory factor analysis supports the construct validity of the scale.

Facility accessibility captures the ease with which residents can reach sports facilities. This dimension is measured using both objective and perceived indicators. Objectively, it is operationalized as the distance between respondents' residences and the nearest sports facility (in meters). Subjectively, respondents also rated perceived accessibility using a five-point Likert scale adapted from previous accessibility measurement studies. The final accessibility measure is constructed based on the standardized combination of these indicators. Higher values indicate better accessibility and lower spatial-temporal barriers to facility use.

#### 3.2.2 Control variable definition

In addition to the independent variables, a set of control variables is introduced to more accurately capture the effects of other factors on individuals' physical activity behavior. These control variables include individual characteristics, social environment factors, and health awareness.

Individual characteristics include age, gender, occupation, income level, and educational attainment. Age is measured as a continuous variable based on respondents' actual chronological age and coded according to its numerical value. Gender is coded as a dummy variable, with males assigned a value of 1 and females assigned a value of 0. Occupation is categorized into five groups, including civil servants, enterprise employees, self-employed individuals, retirees, and students, and is coded using dummy variables (with one category used as the reference group). Income level is classified into three categories (low, middle, and high) according to local average income levels and coded as 1, 2, and 3, respectively. Educational attainment is divided into five levels, including primary school or below, junior secondary school, senior secondary school, junior college, and bachelor's degree or above, and coded from 1 to 5. These individual characteristics may influence exercise behavior and demand for sports facilities; controlling for them helps improve the accuracy of the empirical results.

Social environment factors encompass the family exercise atmosphere and the organization of community sports activities. The family exercise atmosphere is measured using a multi-item Likert scale adapted from previous studies on social support and physical activity environment. Specifically, respondents evaluated family members' exercise habits and the level of encouragement for physical activity on a five-point scale (1 = very poor atmosphere, 5 = very positive atmosphere). The final score is calculated as the mean of all items. Reliability and validity tests indicate acceptable measurement quality, with Cronbach's alpha exceeding 0.70. Community sports activity organization is measured by the number of sports activities organized by the community within a given period (e.g., per month), which is treated as a continuous variable.

Health awareness is measured using a multi-item Likert scale adapted from established literature on health behavior and physical activity awareness. Respondents rated their agreement with statements such as “Physical activity is very important for health" and “Regular exercise can effectively improve health" on a five-point Likert scale (1 = strongly disagree, 5 = strongly agree). The final health awareness score is computed as the average of all items. Cronbach's alpha values exceed 0.70, indicating good internal consistency, and confirmatory factor analysis supports construct validity. Higher scores indicate stronger awareness of the importance of health and physical activity.

Overall, social environment factors play an important role in shaping physical activity behavior, as supportive family environments and frequent community activities can encourage greater participation in exercise. Health awareness represents an internal motivational factor influencing physical activity behavior; individuals with higher levels of health awareness are more likely to engage in regular physical activity.

#### 3.2.3 Dependent variable definition

The dependent variables in this study focus on indicators of individuals' physical activity behavior and mainly include exercise frequency, exercise intensity, and exercise duration.

Exercise frequency refers to the number of times individuals participate in physical activity within a given time period. Data are collected through questionnaire surveys by asking respondents how often they engage in physical activity on a weekly or monthly basis, following commonly used self-reported physical activity measurement approaches in the literature. For example, if an individual exercises three times per week, the exercise frequency is recorded as three. Exercise frequency is treated as a single-item continuous indicator of physical activity engagement; higher frequency generally reflects more active participation in exercise and a stronger emphasis on health.

Exercise intensity describes the level of physical exertion experienced during physical activity. It can be classified based on indicators such as heart rate, energy expenditure, or the vigor of the activity. In this study, exercise intensity is self-reported and categorized into three levels: low intensity (e.g., walking and Tai Chi), moderate intensity (e.g., jogging and cycling), and high intensity (e.g., basketball games and long-distance running), which are coded as 1, 2, and 3, respectively. This is a single-item ordinal measure commonly used in physical activity surveys to capture relative intensity differences across individuals.

Exercise duration refers to the length of time spent on each exercise session. This variable is measured through self-reported responses, asking participants to report the duration of their typical exercise sessions in minutes. For example, if an individual exercises for 60 min per session, the exercise duration is recorded as 60. Exercise duration is treated as a single-item continuous variable widely used in physical activity research to reflect time-based engagement in exercise.

### 3.3 Model establishment

To quantitatively examine the impact of sports facility configuration on individuals' physical activity behavior, this study constructs a multivariate linear regression model. It should be noted that multivariate linear regression is widely applied in social science research, as it allows for an effective analysis of the combined effects of multiple independent variables on a single dependent variable. By estimating the parameters of the model, the quantitative relationships between each independent variable and the dependent variable as well as control variable can be identified, thereby revealing the underlying relationships among the influencing factors.

As stated in Section 3.1, the total sample size is *N* = 1, 800, where *i* = 1, 2, …, 1, 800 indexes individual respondents. Individual physical activity behavior is observed at the individual level and measured along three dimensions: exercise frequency, exercise intensity, and exercise duration. Let *Y*_*k, i*_ denote the observed value of the *k*-th physical activity outcome for individual *i*. Specifically, *Y*_1, *i*_ represents the exercise frequency of individual *i*, *Y*_2, *i*_ represents the exercise intensity of individual *i*, and *Y*_3, *i*_ represents the exercise duration of individual *i*.

At the individual level, the baseline regression models are specified as follows:

$$\begin{array}{l} Y_{1,i}=\beta_{0}+\overset{4}{\underset{j=1}{\sum }}\beta_{j}X_{j,i}+\overset{5}{\underset{m=1}{\sum }}\gamma_{m}C_{1m,i}+\overset{2}{\underset{n=1}{\sum }}\delta_{n}C_{2n,i}+\lambda C_{3,i}+\varepsilon_{1,i}, \end{array}$$ (1)

$$\begin{array}{l} Y_{2,i}=\alpha_{0}+\overset{4}{\underset{j=1}{\sum }}\alpha_{j}X_{j,i}+\overset{5}{\underset{m=1}{\sum }}\theta_{m}C_{1m,i}+\overset{2}{\underset{n=1}{\sum }}\kappa_{n}C_{2n,i}+\mu C_{3,i}+\varepsilon_{2,i}, \end{array}$$ (2)

$$\begin{array}{l} Y_{3,i}=\phi_{0}+\overset{4}{\underset{j=1}{\sum }}\phi_{j}X_{j,i}+\overset{5}{\underset{m=1}{\sum }}\psi_{m}C_{1m,i}+\overset{2}{\underset{n=1}{\sum }}\omega_{n}C_{2n,i}+\xi C_{3,i}+\varepsilon_{3,i}. \end{array}$$ (3)

where ε_*k, i*_ (*k* = 1, 2, 3) denotes the individual-specific stochastic disturbance term capturing unobserved factors affecting exercise frequency, exercise intensity, and exercise duration, respectively. It is assumed that *E*(ε_*k, i*_∣*X*_*i*_, *C*_*i*_) = 0, ensuring unbiased estimation under ordinary least squares (OLS).

Sports facility configuration variables are measured for each individual. Let *X*_*j, i*_ denote the value of the *j*-th sports facility configuration variable for individual *i*, where *j* = 1 corresponds to facility diversity, *j* = 2 to facility quantity, *j* = 3 to facility quality, and *j* = 4 to facility accessibility. The sample mean of each facility-related variable is defined as

$$\begin{array}{l} {\bar{X}}_{j}=\frac{1}{1800}\overset{1800}{\underset{i=1}{\sum }}X_{j,i},\;j=1,2,3,4. \end{array}$$ (4)

Control variables include individual characteristics, social environment factors, and health awareness. Let *C*_1*m, i*_ denote the *m*-th individual characteristic of individual *i*, where *m* = 1 represents age, *m* = 2 represents gender, *m* = 3 represents occupation, *m* = 4 represents income level, and *m* = 5 represents educational attainment. The sample mean of each individual characteristic is defined as

$$\begin{array}{l} {\bar{C}}_{1,m}=\frac{1}{1800}\overset{1800}{\underset{i=1}{\sum }}C_{1,m,i},\;m=1,\ldots ,5. \end{array}$$ (5)

Let *C*_2, *n, i*_ denote the *n*-th social environment variable for individual *i*, where *n* = 1 indicates the frequency of organized physical activities and *n* = 2 indicates the family exercise atmosphere. Based on that, the sample means of social environment variables are defined as

$$\begin{array}{l} {\bar{C}}_{2,n}=\frac{1}{1800}\overset{1800}{\underset{i=1}{\sum }}C_{2,n,i},\;n=1,2. \end{array}$$ (6)

Let *C*_3, *i*_ denote the health awareness level of individual *i*, defined as the individual's cognition, attitudes, and attention toward health-related behaviors. The corresponding sample mean is defined as

$$\begin{array}{l} {\bar{C}}_{3}=\frac{1}{1800}\overset{1800}{\underset{i=1}{\sum }}C_{3,i}. \end{array}$$ (7)

Based on the sample-mean variables defined above, the mean-level representations can be obtained by aggregating the individual-level models above. The sample means of outcomes are defined as

$$\begin{array}{l} {\bar{Y}}_{k}=\frac{1}{1800}\overset{1800}{\underset{i=1}{\sum }}Y_{k,i},\;k=1,2,3. \end{array}$$ (8)

In addition, since *Y*_1, *i*_ (exercise frequency) is a count variable and *Y*_2, *i*_ (exercise intensity) is an ordinal variable, OLS is used as a baseline linear approximation for comparability, while robust standard errors are reported to address potential heteroskedasticity and distributional concerns.

These models, shown in Equations 1–8, are specified at the sample-mean level. Individual-level stochastic disturbance terms are averaged out through aggregation, and their expected values are assumed to be zero. Therefore, no explicit error term is included in the mean-based regression equations.

## 4 Empirical analysis of the effects of multidimensional determinants on physical activity behavior

### 4.1 Descriptive analysis

Table 1 reports the descriptive statistics of the key variables used in the empirical analysis. Regarding sports facility characteristics, the average facility diversity is 3.25, indicating that respondents are generally exposed to multiple types of sports facilities. The mean facility quantity is 15.68, with values ranging from 3 to 50, reflecting substantial variation in facility supply across individuals. Facility quality has an average score of 3.12 on a five point scale, suggesting a moderate overall evaluation by respondents. In terms of accessibility, the average reported distance to sports facilities is 0.85 km, implying relatively convenient access for most respondents in the sample.

**Table 1:** Descriptive statistics of key variables.

Indicator	Symbol	Mean	Max	Min
Facility diversity (count)	*X*_1, *i*_	3.25	7	1
Facility quantity (count)	*X*_2, *i*_	15.68	50	3
Facility quality (5-point scale)	*X*_3, *i*_	3.12	5	1
Facility accessibility (distance, km)	*X*_4, *i*_	0.85	3	0.1
Exercise frequency (times/week)	*Y*_1, *i*_	3.15	8	0
Exercise intensity (5-point scale)	*Y*_2, *i*_	1.86	5	1
Exercise duration (minutes)	*Y*_3, *i*_	45	135	8
Age (years)	*C*_1, 1, *i*_	35.6	70	18
Gender (dummy variable)	*C*_1, 2, *i*_	0.52	1	0
Occupation (dummy variable)	*C*_1, 3, *i*_	1.97	4	0
Income level (3-point scale)	*C*_1, 4, *i*_	2.00	3	1
Educational attainment (5-point scale)	*C*_1, 5, *i*_	3.25	5	1
Family exercise atmosphere (5-point scale)	*C*_2, 2, *i*_	3.20	5	1
Frequency of organized community sports activities (times/month)	*C*_2, 1, *i*_	2.50	5	0
Health awareness (5-point scale)	*C*_3, *i*_	3.50	5	1

With respect to physical activity behavior, individuals exercise on average 3.15 times per week. Exercise intensity is relatively low, with a mean value of 1.86 on a five-point scale, whereas the average exercise duration is 45 min per session, indicating that respondents tend to engage in moderate-duration but low-intensity physical activities.

For control variables, the average age of respondents is 35.6 years, covering a wide age range from 18 to 70. The mean value of the gender dummy variable is 0.52, suggesting a relatively balanced gender distribution. Income level and educational attainment show moderate average values of 2.00 and 3.25, respectively. Social environment variables indicate that respondents participate in organized community sports activities about 2.5 times per month on average, and the family exercise atmosphere scores 3.20 on a five-point scale. Health awareness is relatively high, with an average score of 3.50, implying a generally strong cognition and concern for health-related behaviors among respondents.

### 4.2 Correlation analysis between sports facility configuration and physical activity behavior

**Figure 4:**
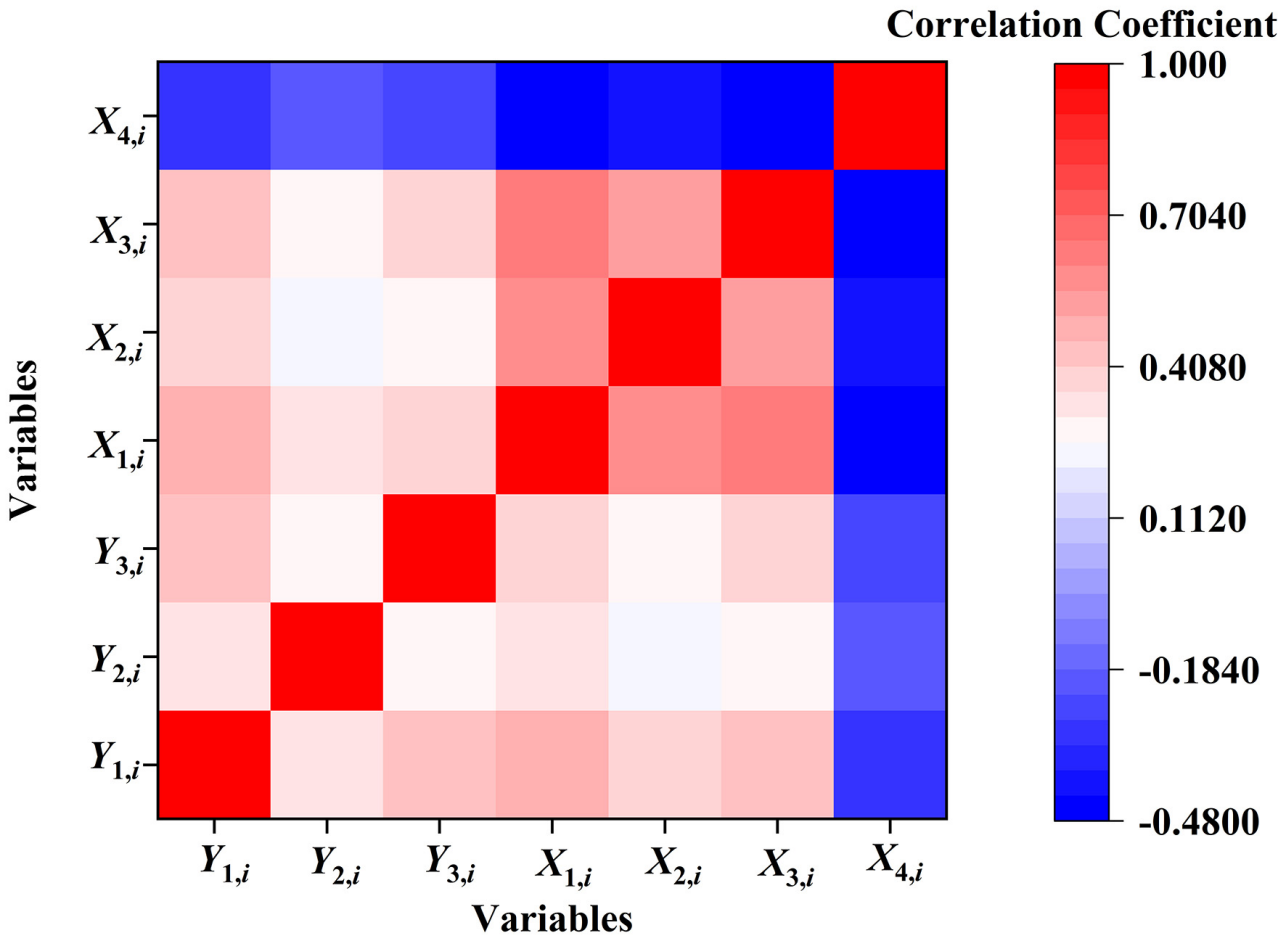
Correlation matrix between sports facility configuration and physical activity behavior.

Table 2 reports the pairwise correlations between sports facility configuration variables and three dimensions of physical activity behavior, and the corresponding correlation matrix between sports facility configuration and physical activity behavior is illustrated in Figure 4. The results show that exercise frequency, exercise intensity, and exercise duration are all positively and significantly correlated with each other, indicating that physical activity behavior exhibits internal consistency across different dimensions. Individuals who exercise more frequently also tend to engage in longer and more intensive exercise sessions, reflecting the habitual and reinforcing nature of physical activity behavior.

**Table 2:** Correlation analysis between sports facility configuration and physical activity behavior.

Variable	*Y*_1, *i*_	*Y*_2, *i*_	*Y*_3, *i*_	*X*_1, *i*_	*X*_2, *i*_	*X*_3, *i*_	*X*_4, *i*_
*Y*_1, *i*_ Exercise frequency	1	0.356^***^	0.421^***^	0.485^***^	0.397^***^	0.453^***^	−0.327^***^
*Y*_2, *i*_ Exercise intensity	0.356^***^	1	0.289^***^	0.324^***^	0.258^***^	0.306^***^	−0.215^***^
*Y*_3, *i*_ Exercise duration	0.421^***^	0.289^***^	1	0.402^***^	0.305^***^	0.387^***^	−0.274^***^
*X*_1, *i*_ Facility diversity	0.485^***^	0.324^***^	0.402^***^	1	0.563^***^	0.621^***^	−0.436^***^
*X*_2, *i*_ Facility quantity	0.397^***^	0.258^***^	0.305^***^	0.563^***^	1	0.514^***^	−0.382^***^
*X*_3, *i*_ Facility quality	0.453^***^	0.306^***^	0.387^***^	0.621^***^	0.514^***^	1	−0.478^***^
*X*_4, *i*_ Facility accessibility	−0.327^***^	−0.215^***^	−0.274^***^	−0.436^***^	−0.382^***^	−0.478^***^	1

^***^indicates statistical significance at the 1% level.

Regarding sports facility configuration, facility diversity, facility quantity, and facility quality are all positively and significantly correlated with exercise frequency, intensity, and duration. These findings suggest that a more diverse, abundant, and higher quality sports facility environment is associated with greater participation in physical activity and more favorable exercise patterns. From the perspective of health promotion theory, this pattern is consistent with the role of supportive physical environments in facilitating healthy behaviors. Diverse and high quality facilities increase the availability of exercise opportunities and are associated with greater attractiveness of physical activity, which may be linked to individuals' higher likelihood of initiating and maintaining regular exercise.

Facility accessibility, measured in terms of distance or time cost, is negatively and significantly correlated with all three dimensions of physical activity behavior. Given that accessibility is operationalized as the distance or travel time to the nearest sports facility, this negative association indicates that individuals facing greater spatial or temporal barriers tend to exhibit lower exercise frequency, reduced exercise intensity, and shorter exercise duration. This pattern is broadly consistent with behavior change theory, which emphasizes that higher behavioral costs may reduce the likelihood of behavior adoption and persistence. As the perceived or actual cost of accessing facilities increases, individuals become less likely to engage in physical activity, even when sufficient motivation is present.

Moreover, the strong correlations observed among facility diversity, quantity, and quality suggest that sports facility configuration dimensions tend to improve simultaneously rather than independently. From a health promotion perspective, such coordinated improvements in the built environment may be associated with multiple supportive conditions for health behaviors, including increased opportunity, enhanced perceived benefits, and improved exercise experiences.

Overall, the correlation analysis provides preliminary and descriptive evidence consistent with the theoretical framework derived from health promotion and behavior change theories. It highlights the importance of sports facility configuration as a potential environmental factor associated with physical activity behavior and lays the groundwork for subsequent multivariate regression analysis aimed at examining the relationships between facility related factors and physical activity behavior while controlling for individual characteristics, social environment, and health awareness.

### 4.3 Regression analysis

Table 3 presents the empirical regression results on the associations between multidimensional determinants and physical activity behavior. As the analysis is based on cross-sectional survey data, the estimated coefficients are interpreted as statistical associations rather than causal effects. From the perspective of health promotion and behavior change theories, the results indicate that facility-related characteristics, including facility type diversity (${\bar{X}}_{1}$), facility quantity (${\bar{X}}_{2}$), facility quality (${\bar{X}}_{3}$), and facility accessibility (${\bar{X}}_{4}$), are significantly associated with individual physical activity behavior, while individual characteristics, social environment factors, and health awareness are included as control variables. Specifically, facility diversity, quantity, and quality are positively and significantly associated with exercise frequency (${\bar{Y}}_{1}$), exercise intensity (${\bar{Y}}_{2}$), and exercise duration (${\bar{Y}}_{3}$). These associations suggest that a more diversified, sufficient, and higher-quality built environment is correlated with higher levels of physical activity, which is consistent with ecological models of health promotion. In contrast, the negative association between facility accessibility and all three dimensions of physical activity should be interpreted in light of its measurement as distance rather than accessibility *per se*. The results indicate that greater distance to sports facilities is associated with lower physical activity participation. In dense urban contexts, longer effective travel distances or time costs may reflect not only physical separation but also congestion, fragmented urban layouts, or competition for facility use, which are associated with lower levels of regular and intensive exercise. These interpretations are consistent with insights from behavioral economics and habit-formation theories, which emphasize the role of access costs and friction in shaping daily activity choices.

**Table 3:** Regression results of multidimensional determinants on physical activity behavior.

Variable	Exercise frequency (${\bar{Y}}_{1}$)	Exercise intensity (${\bar{Y}}_{2}$)	Exercise duration (${\bar{Y}}_{3}$)
Facility diversity (${\bar{X}}_{1}$)	0.352^***^ (0.042)	0.205^***^ (0.035)	0.286^***^ (0.038)
Facility quantity (${\bar{X}}_{2}$)	0.236^***^ (0.031)	0.128^***^ (0.025)	0.165^***^ (0.028)
Facility quality (${\bar{X}}_{3}$)	0.289^***^ (0.035)	0.156^***^ (0.028)	0.213^***^ (0.032)
Facility accessibility (${\bar{X}}_{4}$)	−0.187^***^ (0.028)	−0.102^***^ (0.022)	−0.134^***^ (0.025)
Age (${\bar{C}}_{1,1}$)	−0.012^**^ (0.005)	−0.008^*^ (0.004)	−0.010^**^ (0.004)
Gender (Male = 1, Female = 0) (${\bar{C}}_{1,2}$)	0.156^***^ (0.038)	0.085^***^ (0.030)	0.102^***^ (0.033)
Enterprise employees (${\bar{C}}_{1,3}^{1}$)	−0.085^**^ (0.035)	−0.042 (0.028)	−0.056^*^ (0.031)
Self-employed (${\bar{C}}_{1,3}^{2}$)	−0.102^***^ (0.036)	−0.058^*^ (0.029)	−0.075^**^ (0.032)
Retirees (${\bar{C}}_{1,3}^{3}$)	0.098^***^ (0.034)	0.056^*^ (0.027)	0.072^**^ (0.030)
Students (${\bar{C}}_{1,3}^{4}$)	−0.125^***^ (0.037)	−0.068^**^ (0.030)	−0.090^***^ (0.033)
Income level (${\bar{C}}_{1,4}$)	0.075^***^ (0.024)	0.041^*^ (0.019)	0.053^**^ (0.021)
Educational attainment (${\bar{C}}_{1,5}$)	0.056^**^ (0.023)	0.030 (0.018)	0.042^*^ (0.020)
Community physical activity organization (${\bar{C}}_{2,1}$)	0.086^***^ (0.025)	0.048^**^ (0.020)	0.063^***^ (0.022)
Family exercise atmosphere (${\bar{C}}_{2,2}$)	0.123^***^ (0.029)	0.068^***^ (0.023)	0.095^***^ (0.026)
Health awareness (${\bar{C}}_{3}$)	0.152^***^ (0.027)	0.082^***^ (0.022)	0.110^***^ (0.024)
Constant	1.256^***^ (0.152)	0.823^***^ (0.121)	1.035^***^ (0.135)
Sample size	1,800	1,800	1,800
*R* ^2^	0.456	0.328	0.387

Robust standard errors are reported in parentheses. ^***^, ^**^, and ^*^ indicate statistical significance at the 1%, 5%, and 10% levels, respectively. Female respondents and civil servants are used as the reference categories for gender and occupation dummy variables.

Regarding individual characteristics, age ($\bar{C}1,1$) is negatively and significantly associated with physical activity participation, reflecting life-course constraints on activity engagement, while gender ($\bar{C}1,2$) shows a positive and significant association, indicating higher reported physical activity among males, with females as the reference group. Occupational heterogeneity further indicates that, relative to civil servants (the reference category), enterprise employees, self-employed individuals, and students are associated with lower levels of physical activity participation, whereas retirees are associated with higher participation. Moreover, social environment factors, including community physical activity organization ($\bar{C}2,1$) and family exercise atmosphere ($\bar{C}2,2$), are positively associated with physical activity outcomes, highlighting the relevance of social support and normative influences emphasized in behavior change theories. Health awareness (${\bar{C}}_{3}$) is consistently and positively associated with all physical activity outcomes, suggesting that individuals' health-related cognition and attitudes are closely related to exercise behavior. Collectively, these results indicate that physical activity behavior is associated with a combination of facility-related characteristics, social environmental factors, and individual health awareness.

### 4.4 Heterogeneity test

To further explore heterogeneity in the associations between sports facility configuration and physical activity behavior across population groups, this study conducts subgroup analyses along three dimensions: age, gender, and income level. In each subgroup regression, the same set of control variables as in the baseline model is included to ensure comparability of the estimated coefficients across groups. Given the cross-sectional nature of the data, the following results are interpreted as heterogeneous associations rather than causal effects.

Table 4 presents the heterogeneous associations by age group. The results indicate that the associations between sports facility configuration and physical activity outcomes vary systematically across life stages. Facility diversity (${\bar{X}}_{1}$), quantity (${\bar{X}}_{2}$), and quality (${\bar{X}}_{3}$) show the strongest positive associations with exercise frequency, intensity, and duration among younger individuals, with coefficient magnitudes gradually declining for middle-aged and older groups. This pattern is consistent with ecological models of health behavior, which suggest that younger individuals may be more responsive to environmental opportunity structures due to greater physical capacity and behavioral flexibility. By contrast, the relatively weaker associations observed among older adults suggest that improvements in the built environment alone may be insufficient to substantially increase physical activity without complementary measures addressing physical limitations and motivational constraints. In addition, facility accessibility (${\bar{X}}_{4}$), measured as the distance or time cost to the nearest sports facility, exhibits a consistently negative association with physical activity across all age groups, indicating that greater distance is associated with lower levels of participation. This association appears more pronounced among older individuals, who may be more sensitive to travel burden, environmental complexity, and safety concerns. Overall, these patterns highlight the importance of age-sensitive facility planning and life-course-oriented physical activity promotion strategies that reduce effective access costs.

**Table 4:** Heterogeneous effects by age group.

Variable	YF-Y	YF-M	YF-O	YI-Y	YI-M	YI-O	YD-Y	YD-M	YD-O
Facility diversity (${\bar{X}}_{1}$)	0.425^***^	0.306^***^	0.258^***^	0.286^***^	0.182^***^	0.135^***^	0.356^***^	0.245^***^	0.201^***^
Facility quantity (${\bar{X}}_{2}$)	0.285^***^	0.202^***^	0.156^***^	0.165^***^	0.105^***^	0.082^***^	0.205^***^	0.138^***^	0.108^***^
Facility quality (${\bar{X}}_{3}$)	0.356^***^	0.253^***^	0.201^***^	0.198^***^	0.136^***^	0.102^***^	0.268^***^	0.185^***^	0.143^***^
Facility accessibility (${\bar{X}}_{4}$)	−0.225^***^	−0.164^***^	−0.128^***^	−0.132^***^	−0.095^***^	−0.072^***^	−0.178^***^	−0.124^***^	−0.098^***^
Constant	0.856^***^	1.456^***^	1.823^***^	0.563^***^	0.925^***^	1.256^***^	0.785^***^	1.123^***^	1.568^***^
Observations	600	600	600	600	600	600	600	600	600
*R* ^2^	0.523	0.412	0.356	0.387	0.289	0.225	0.456	0.345	0.298

YF, YI, and YD denote exercise frequency (${\bar{Y}}_{1}$), intensity (${\bar{Y}}_{2}$), and duration (${\bar{Y}}_{3}$), respectively. Y, M, and O refer to youth, middle-aged, and older groups. ^***^ indicates significance at the 1% level.

Table 5 reports the heterogeneous associations by gender. The results reveal systematic gender differences in how sports facility configuration is associated with physical activity behavior. Across all three outcomes, the coefficients associated with facility diversity (${\bar{X}}_{1}$), quantity (${\bar{X}}_{2}$), and quality (${\bar{X}}_{3}$) are larger for males than for females, suggesting that men may be more likely to translate environmental opportunities into higher exercise frequency, intensity, and duration. This pattern is consistent with gender-related behavior theories emphasizing the role of social norms, perceived competence, and risk tolerance in shaping responsiveness to physical environments. Although the corresponding associations remain statistically significant for females, the smaller magnitudes suggest that additional psychosocial constraints, such as safety concerns, time limitations, or caregiving responsibilities, may attenuate the relationship between facility provision and physical activity. Meanwhile, facility accessibility (${\bar{X}}_{4}$) shows a negative association with physical activity for all genders, indicating that greater distance to sports facilities constitutes a barrier to engagement. These findings suggest that reducing spatial and temporal access costs is a necessary but not sufficient condition for promoting physical activity, and that inclusive design and gender-sensitive programming may be required to translate improved proximity into sustained participation.

**Table 5:** Heterogeneous effects by gender.

Variable	YF-Male	YF-Female	YI-Male	YI-Female	YD-Male	YD-Female
Facility diversity (${\bar{X}}_{1}$)	0.402^***^	0.308^***^	0.256^***^	0.185^***^	0.325^***^	0.236^***^
Facility quantity (${\bar{X}}_{2}$)	0.268^***^	0.205^***^	0.145^***^	0.102^***^	0.186^***^	0.138^***^
Facility quality (${\bar{X}}_{3}$)	0.321^***^	0.246^***^	0.178^***^	0.125^***^	0.234^***^	0.175^***^
Facility accessibility (${\bar{X}}_{4}$)	−0.205^***^	−0.152^***^	−0.115^***^	−0.086^***^	−0.158^***^	−0.112^***^
Constant	1.123^***^	1.356^***^	0.785^***^	0.925^***^	0.956^***^	1.185^***^
Observations	936	864	936	864	936	864
*R* ^2^	0.487	0.398	0.356	0.268	0.423	0.325

Male and female subsamples are estimated separately. ^***^indicates significance at the 1% level.

Table 6 presents the heterogeneous associations by income level. The results demonstrate a clear socioeconomic gradient in the relationship between sports facility configuration and physical activity behavior. The positive associations of facility diversity (${\bar{X}}_{1}$), quantity (${\bar{X}}_{2}$), and quality (${\bar{X}}_{3}$) with all three physical activity outcomes increase monotonically from low-income to high-income groups. This pattern is consistent with theories of behavioral opportunity and resource complementarity, which suggest that individuals with greater economic resources may be better positioned to convert environmental infrastructure into actual health-related behaviors. By contrast, the weaker associations observed among low-income individuals imply that facility provision alone may not fully offset structural barriers such as work intensity, stress, or limited access to supportive social networks. Facility accessibility (${\bar{X}}_{4}$) remains negatively associated with physical activity across income groups, indicating that greater distance is linked to lower participation. Collectively, these findings underscore the importance of integrating facility investment with targeted policy interventions, such as subsidized programs and community engagement initiatives, to promote more equitable physical activity outcomes.

**Table 6:** Heterogeneous effects by income level.

Variable	YF-L	YF-M	YF-H	YI-L	YI-M	YI-H	YD-L	YD-M	YD-H
Facility diversity (${\bar{X}}_{1}$)	0.286^***^	0.365^***^	0.452^***^	0.165^***^	0.225^***^	0.286^***^	0.225^***^	0.306^***^	0.385^***^
Facility quantity (${\bar{X}}_{2}$)	0.185^***^	0.245^***^	0.302^***^	0.098^***^	0.135^***^	0.178^***^	0.128^***^	0.176^***^	0.225^***^
Facility quality (${\bar{X}}_{3}$)	0.221^***^	0.298^***^	0.365^***^	0.115^***^	0.168^***^	0.213^***^	0.156^***^	0.214^***^	0.278^***^
Facility accessibility (${\bar{X}}_{4}$)	−0.152^***^	−0.187^***^	−0.225^***^	−0.086^***^	−0.102^***^	−0.132^***^	−0.112^***^	−0.134^***^	−0.178^***^
Constant	1.456^***^	1.235^***^	0.986^***^	1.023^***^	0.856^***^	0.623^***^	1.235^***^	1.086^***^	0.856^***^
Observations	540	900	360	540	900	360	540	900	360
*R* ^2^	0.356	0.432	0.508	0.225	0.306	0.387	0.298	0.378	0.456

L, M, and H denote low-, middle-, and high-income groups. ^***^ indicates significance at the 1% level.

### 4.5 Robustness test

To examine the robustness of the baseline regression results, this study conducts a series of robustness checks by replacing the original facility-related explanatory variables with alternative but conceptually related measures. Throughout these analyses, the dependent variables, including exercise frequency, exercise intensity, and exercise duration, remain unchanged, and the same set of control variables is consistently included across all model specifications. If the estimated coefficients of the core facility-related variables remain statistically significant and retain consistent signs after variable substitution, the observed associations can be considered robust to alternative variable definitions. This approach helps mitigate concerns regarding measurement bias and reduces the likelihood that the results are driven by a specific operationalization of facility characteristics.

Table 7 reports the robustness check results using alternative measures of sports facility characteristics. Consistent with the baseline results, the coefficients of facility diversity (${\bar{X}}_{1}$), facility quantity (${\bar{X}}_{2}$), and facility condition (${\bar{X}}_{3}$) remain statistically significant and exhibit stable positive associations with exercise frequency (${\bar{Y}}_{1}$), exercise intensity (${\bar{Y}}_{2}$), and exercise duration (${\bar{Y}}_{3}$). These findings suggest that the observed positive associations between sports facility configuration and physical activity behavior are not sensitive to specific variable definitions, but instead reflect a relatively stable empirical relationship. Meanwhile, facility accessibility (${\bar{X}}_{4}$), measured as the distance or time cost to the nearest sports facility, consistently shows a negative association with all three physical activity outcomes. This indicates that greater spatial or temporal distance is associated with lower exercise frequency, intensity, and duration. In dense urban environments, longer effective access distances may capture not only physical separation but also congestion, fragmented urban layouts, or competition for facility use, all of which increase behavioral costs and are associated with reduced participation in physical activity.

**Table 7:** Robustness check results using alternative facility measures.

Variable	Exercise frequency (${\bar{Y}}_{1}$)	Exercise intensity (${\bar{Y}}_{2}$)	Exercise duration (${\bar{Y}}_{3}$)
Facility diversity (${\bar{X}}_{1}$)	0.348^***^ (0.043)	0.202^***^ (0.036)	0.282^***^ (0.039)
Facility quantity (${\bar{X}}_{2}$)	0.234^***^ (0.032)	0.126^***^ (0.026)	0.162^***^ (0.029)
Facility condition (${\bar{X}}_{3}$)	0.278^***^ (0.037)	0.152^***^ (0.030)	0.208^***^ (0.033)
Facility accessibility (${\bar{X}}_{4}$)	−0.185^***^ (0.029)	−0.101^***^ (0.023)	−0.132^***^ (0.026)
Constant	1.248^***^ (0.154)	0.816^***^ (0.123)	1.028^***^ (0.137)
Observations	1,800	1,800	1,800
*R* ^2^	0.452	0.324	0.385

Robust standard errors are reported in parentheses. ^***^ indicates statistical significance at the 1% level.

Overall, while these robustness checks do not establish causal effects, they provide additional support for the stability of the observed associations between the built environment and physical activity behavior. In line with ecological and behavior change frameworks, the results underscore that physical activity participation is closely related to environmental affordances and access-related constraints, thereby strengthening confidence in the empirical patterns identified in this study.

## 5 Policy implications

Based on the empirical findings, several policy implications can be drawn. First, governments should play a leading role in strengthening policy support and financial investment in sports facility development. Guided by the ecological model of health promotion, facility planning should consider local population density, age structure, and socioeconomic conditions to ensure a balanced and equitable spatial distribution of facilities, with particular attention to increasing facility diversity, quantity, and quality in densely populated urban areas and newly developed communities.

Second, facility construction should emphasize diversification and differentiated design to better accommodate the needs of different population groups. In line with behavior change theories emphasizing person-environment fit, competitive and multifunctional facilities can be prioritized for younger populations, while safe, comfortable, and age-friendly facilities should be provided for older adults. Meanwhile, strict quality standards, regular maintenance, and effective management mechanisms are essential to ensure facility safety and long-term usability.

Third, enhanced public communication and health promotion efforts are necessary to improve residents' awareness of available facilities and the benefits of physical activity. By integrating media outreach, community-based programs, and organized sports activities, policymakers can strengthen health awareness and social support, thereby activating key motivational mechanisms highlighted in the HBM and the Theory of Planned Behavior.

Overall, the findings suggest that promoting physical activity requires the coordinated optimization of physical infrastructure, social environments, and individual-level health cognition. Such an integrated, theory-informed approach is crucial for improving facility utilization, encouraging sustained behavior change, and advancing the high-quality development of national fitness initiatives.

## 6 Conclusions

Using questionnaire survey data, this study empirically examines the effects of multidimensional determinants, including facility-related characteristics, individual characteristics, social environment factors, and health awareness, on physical activity behavior through multiple statistical methods. Consistent with findings from previous studies on built environment and physical activity, as well as health promotion and ecological behavior change theories, the results indicate that facility type diversity, facility quantity, and facility quality are all significantly and positively associated with exercise frequency, intensity, and duration. This supports the view in existing literature that a well-developed and diversified sports facility environment can reduce structural barriers and increase behavioral opportunities for physical activity. From a theoretical perspective, these findings further extend ecological models of health behavior by empirically confirming that multiple dimensions of facility provision jointly contribute to shaping exercise behavior, rather than operating through a single environmental attribute.

In contrast, facility accessibility shows a significant negative association with physical activity behavior. This finding is consistent with cost-related explanations in behavioral economics and habit-formation theories, which emphasize that distance- and time-related frictions can reduce the likelihood of sustained participation in health-related behaviors. One possible explanation is that higher travel costs may discourage regular engagement in physical activity, particularly in contexts where alternative low-effort activities are available, thereby weakening overall exercise participation.

Moreover, the effects of sports facility configuration exhibit clear heterogeneity across population groups. The associations are generally stronger among younger individuals, males, and higher-income groups. This pattern may be explained by differences in time availability, opportunity costs, and behavioral responsiveness across social and demographic groups. In particular, individuals with fewer time constraints and greater discretionary resources may be better able to translate environmental opportunities into actual physical activity, whereas those facing higher life or work pressures may be less responsive to improvements in facility conditions. These findings further complement behavior change theories by highlighting the importance of differentiated and targeted interventions across population subgroups.

Finally, several limitations should be acknowledged. First, the cross-sectional nature of the data limits the ability to establish causal relationships, and therefore all findings are interpreted as statistical associations. Second, physical activity measures and some explanatory variables rely on self-reported questionnaire data, which may introduce recall bias and reporting bias, as well as potential measurement error. Third, although the study covers five representative Chinese cities, regional differences in socioeconomic development and sports infrastructure may still limit the external validity of the findings. In addition, potential clustering effects at the community or city level may exist, which are not fully accounted for in the current analysis and could influence the precision of the estimated associations. Future research could benefit from longitudinal designs, objective physical activity measurement tools, multilevel modeling approaches to address clustering structures, and broader geographic coverage to further validate and extend these results.

## Data Availability

The raw data supporting the conclusions of this article will be made available by the authors, without undue reservation.
